# High-intensity focused ultrasound ablation combined with immunotherapy for treating liver metastases: A prospective non-randomized trial

**DOI:** 10.1371/journal.pone.0306595

**Published:** 2024-07-05

**Authors:** Xiyue Yang, Yao Liao, Lingli Fan, Binwei Lin, Jie Li, Danfeng Wu, Dongbiao Liao, Li Yuan, Jihui Liu, Feng Gao, Gang Feng, Xiaobo Du

**Affiliations:** 1 Department of Oncology, Mianyang Central Hospital, Mianyang, China; 2 State Key Laboratory of Ultrasound in Medicine and Engineering, College of Biomedical Engineering, Chongqing Medical University, Chongqing, China; 3 Sichuan Clinical Research Center for Radiation and Therapy, Mianyang, China; 4 Chongqing Key Laboratory of Biomedical Engineering, Chongqing Medical University, Chongqing, China; UT Austin: The University of Texas at Austin, UNITED STATES

## Abstract

**Purpose:**

Given the unique features of the liver, it is necessary to combine immunotherapy with other therapies to improve its efficacy in patients of advanced cancer with liver metastases (LM). High-intensity focused ultrasound (HIFU) ablation is now widely used in clinical practice and can enhanced immune benefits. The study is intended to prospectively evaluate the safety and clinical feasibility of HIFU ablation in combination with systemic immunotherapy for patients with liver metastases.

**Methods:**

The study enrolled 14 patients with LM who received ultrasound-guided HIFU ablation combined with immune checkpoint inhibitors (ICIs) such as anti-programmed cell death protein 1 (anti-PD-1 agents manufactured in China) at Mianyang Central Hospital. Patients were followed up for adverse events (AEs) during the trial, using the CommonTerminology Criteria for Adverse Events v5.0(CTCAE v5.0) as the standard. Tumour response after treatment was assessed using computerized tomography.

**Results:**

The 14 patients (age range, 35–84 years) underwent HIFU ablation at 19 metastatic sites and systemic immunotherapy. The mean lesion volume was 179.9 cm3 (maximum: 733.1 cm3). Median follow-up for this trial was 9 months (range: 3–21) months. The study is clinically feasible and acceptable to patients.

**Conclusion:**

This prospective study confirmed that HIFU combined with immunotherapy is clinically feasible and safe for treating liver metastases.

## 1. Introduction

Cancer metastases are the causes of over 90% of mortalities associated with advanced solid tumor [1, 2]. The liver has rich hemodynamic features (both portal venous and arterial systems) and unique microenvironment which render it intrinsically susceptible to disseminated tumor cells, resulting in a metastasis rate of 11.1%, one of the most common targets for metastasis [3, 4]. The incidence of primary malignancies and liver metastases (LM) has increased in recent years [5]. Approximately 40% of patients with malignant tumors develop LM [6], which greatly impacts patient survival [4]. Treatment involves two aspects: primary tumors and LM [7, 8]. If these cannot be radically resected by surgery, it is difficult to control the progression of advanced cancer in the long term, even with the wide range of current treatment options [9]. Therefore, effective and less toxic combination therapies need to be actively explored for patients with liver metastases, especially those after multiple lines of treatment.

The advent of immunotherapy has achieved great success in clinical practice and has gradually moved from being a popular new treatment to a first-line recommendation in guidelines for many cancers [10–14]. To date, the US FDA has approved various immunotherapeutic agents, of which the most widely used in clinical is anti-PD1-PDL1 [11, 15–18]. They have been routinely used in the treatment of common malignancies owing to their favorable toxicity profile, clinical benefit, and patients’ quality of life [19, 20]. However, the presence of liver metastases in patients with advanced cancer will lead to a lack of response to immunotherapy, an immunosuppressive effect that has been demonstrated in several studies by modulating and activating systemic and intra-tumoural immune cells [21]. In addition, macrophage-induced apoptosis eliminates tumor-specific CD8+ T cells, thereby promoting hepatic immunetolerance [22]. As such, although some studies have shown that ICI-based immunotherapy improves overall survival in patients with advanced cancer, patients with liver metastases achieve less overall benefit [23]. Therefore, it is necessary to combine immunotherapy with other therapies to achieve synergistic effects by reversing the immunosuppressive tumor microenvironment [24–27].

ICI-based immunotherapy in conjunction with cytotoxic chemotherapy have been widely used as the standard clinical treatment [28]. Clinical trial data (Impower150) has suggested that chemotherapy may enhance the efficacy of ICIs in LM patients to some extent [29]. The local effect of radiotherapy in metastatic cancer can stimulate systemic immunity, and radiotherapy combined with immunotherapy is more common in clinical practice [30].

For example, radiotherapy enhanced the systemic effect of immunotherapy, leading to the regression of distant metastatic cancer [31]. Minimally ablative therapies have also shown immunomodulatory effects in patients with LM [32–36], mobilizing systemic immune cells for an anti-tumour immune response by exposing tumour-associated antigens [37]. High-intensity focused ultrasound ablation was originally applied to gynecological benign tumors such as uterine fibroids, and is now widely used in the treatment of advanced and metastatic malignancies because it is a safe, non-interventional therapy [38, 39]. HIFU can accurately treat targeted lesions and produce thermal effects (T-HIFU) that induced coagulative necrosis of the tumour or mechanical effects (M-HIFU) that destroyed the tumour and thus enhanced tumour antigenicity [37, 38, 40–42]. The local therapeutic effects of HIFU ablation in patients have also been demonstrated in a number of study [43–48]. Moreover, extensive clinical and preclinical studies have shown that HIFU thermal ablation induces long-term systemic anti-tumour immunity in the host, in addition to direct tumor destruction [31–36, 49]. Thus, HIFU may enhance the efficacy of immunotherapy in clinical practice.

To date, only two ongoing clinical trials have combined HIFU with anti-PD-1 immunotherapy. Trial registration numbers are: (NCT03237572), (NCT04116320), respectively: HIFU combined with immunotherapy in the treatment of metastatic breast cancer; Focused ultrasound ablation (FUSA) combined with pembrolizumab for solid tumors [50]. To our knowledge, no studies have been conducted combining anti-PD-1 immunotherapy with HIFU ablation for the treatment of liver metastases. Therefore, this study explored the safety and workflow feasibility of combining the two treatments for patients with liver metastases.

## 2. Materials and methods

The study registration date (registration number: ChiCTR2100043123) is available at the Chinese Clinical Trials Registry (date of registration 05/02/2021). Where applicable to the study design, the Transparent Reporting of Evaluations with Non-randomized Designs (TREND) reporting guidelines were followed.

### 2.1. Ethics statement

This study was approved by the Ethics Committee of Mianyang Central Hospital, Sichuan, China (approval number: S-2020-054) and was conducted according to the tenets of the Declaration of Helsinki. Prior to the intervention, the investigator should discuss the associated risks and specific treatment with each patient and the informed written consent form should be formally signed by the subject party.

### 2.2. Safety assessments and efficacy

The primary endpoint is the safety and feasibility of the combination of the two treatments. Adverse events (AEs), such as pain scores and calculated nonperfusion volume (NPV) ratios, were recorded. NPV% is generally used to represent the volume of ablation, which can evaluate the therapeutic effect of HIFU. Secondary endpoints are tumour response and progression, with follow-up review and recording of different rates of tumour size, such as disease control rate (DCR), objective response rate (ORR). All target lesions (including measurable primary lesions, hepatic metastases, and remaining metastatic site lesions) were measured by computed tomography (CT) imaging and initial baseline conditions were recorded, and efficacy was assessed thereafter every two cycles of immunotherapy. Tumour response was assessed against the Response Evaluation Criteria in Solid Tumours (RECIST) version 1.1 compared to baseline. Patients were followed up for adverse events (AEs) during the trial, using the Common Terminology for Adverse Events version 5.0 as the standard.

### 2.3. Patient characteristics

From February 25, 2021 to March 11, 2022, 14 patients with LM who received HIFU treatment combined with immunotherapy at Mianyang Central Hospital were selected for this study. Patients had previously received standard treatment for primary tumors, but after multiple anti-tumor interventions, including targeted therapy, chemotherapy, radiotherapy, and surgery, the patients developed advanced cancers with extensive systemic metastases. All patients were eligible for reoperation, could tolerate systemic chemotherapy, and could undergo multiple radiotherapy sessions at the same site. Eligible patients volunteered to participate in this study after being informed that systemic immunotherapy in combination with HIFU therapy has relatively few toxic side effects and that HIFU therapy can be repeated multiple times. Immunotherapy was administered within 1 month before and after HIFU ablation to be considered a combination of the two treatments. While there are clinical trials investigating the use of HIFU for immunomodulation of malignant tumors, none have specifically examined the combination of HIFU with immunotherapy [50]. Based on relevant preclinical research, the timing of immunotherapy and HIFU treatment is not rigidly defined [52–54]. The initial design and inclusion criteria of our trial aimed to integrate immunotherapy during the period of HIFU treatment (within 1 month before and after HIFU). Given the absence of literature comparing the efficacy and side effects of different immunological drugs in patients with liver metastases, we refrained from selecting a specific immune checkpoint inhibitor.

Inclusion criteria of patients were: aged 18–75 years; ECOG (Eastern Cooperative Oncology Group) performance status scored 0–2 points; the presence of liver metastases confirmed by diagnostic imaging or by needle biopsy; with no contraindications to immunotherapy and those aware of the associated side effects; with a strong desire for treatment, who understood and agreed to the HIFU treatment and the possible risks (including caregivers); agreement to use immunotherapy in combination during HIFU treatment (within 1 month before and after); who were aware of the range of lesions treatable and safety of HIFU technology; with measurable target lesion assessed by RECIST 1.1; survived at least 12 weeks; with normal function of vital organs.

Exclusion criteria are: patients with uncontrolled hypertension or hyperglycaemia or a history of severe unnormal function of vital organs; who were pregnant or lactating; patients with significant scarring of the abdominal wall of the acoustic pathway; who have received prior radiotherapy doses >45 Gy at the same site as the HIFU treatment; with acute abdomen inflammatory disease; with tumors not visible using our monitoring system; with clearly diagnosed hepatic failure (e.g. hepatic encephalopathy or significant ascites); with abnormal coagulation and therapies such as thrombolysis or anticoagulation within 4 weeks of initiating intervention.

### 2.4. Pre-HIFU preparation

All patients undergo specific bowel preparation prior to HIFU ablation by feeding dregs-free liquid diet for 2–3 days, fasting for 10 hours prior to procedure and having an enema the morning before the procedure. Cleansing of the skin around the target lesion to achieve degreasing and degassing is a routine topical skin preparation prior to HIFU treatment. All patients provided samples for immunoassay, thyroid function, adrenocortical hormone, and cardiac marker analyses before initiating immunotherapy. HIFU ablation used model-jc-focused ultrasound tumour treatment system, manufactured by Chongqing HIFU Medical Technology Co Ltd, China. During HIFU treatment, as with all general anaesthetic procedures, a specialist anesthetist is required to ensure that the treatment is carried out under intravenous sedation and analgesia to prevent pain and discomfort, as well as to monitor the patient’s vital signs.

### 2.5. Follow-up

All patients were transferred to ICU, after HIFU treatment, and returned to their original wards from day 2 onwards. For the first 3 days post-treatment, follow-ups to assess the incidence of pain flares and any complications were performed daily. For the first month post-treatment daily, telephone follow-ups were conducted to measure any patient discomfort from day 4. The patients returned to the hospital for follow-up every two months post-treatment for imaging and symptom evaluation.

All AEs were recorded independently of their relationship with the treated metastasis. AEs were classified as absolutely unrelated, probably unrelated, possibly related, probably related or absolutely related according to their relevance to treatment and all AEs were scored by a specialist clinician. The stopping rule will be triggered if three patients develop severe treatment-related AEs, according to the International Organization for Standardization Criteria. Pain and discomfort were measured using the Visual Analogue Scale (VAS), which is self-reported pain assessment tool that uses 10-point scale, with 0 being pain-free gradually increasing to 10 being most painful.

### 2.6 Statistical methods

SPSS 22.0 statistical software was used to analyze the data collected by the cutoff date of March 11, 2022. The objective response rate (ORR) was calculated using the Clopper-Pearson method.

## 3. Results

### 3.1. Patient population

**Fig 1:**
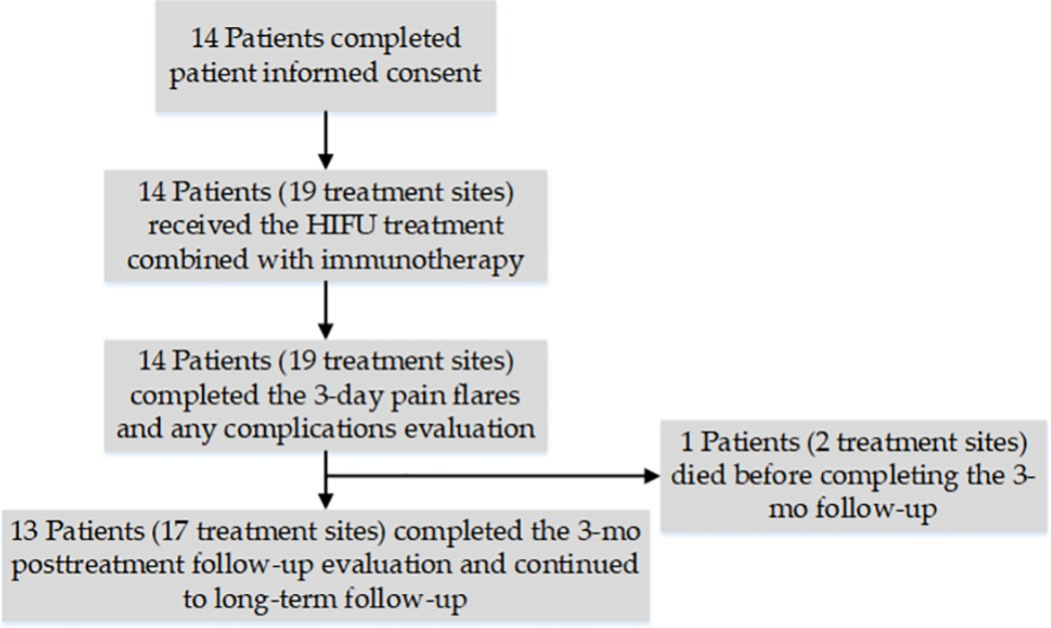
Consort participant flow diagram.

**Fig 2:**
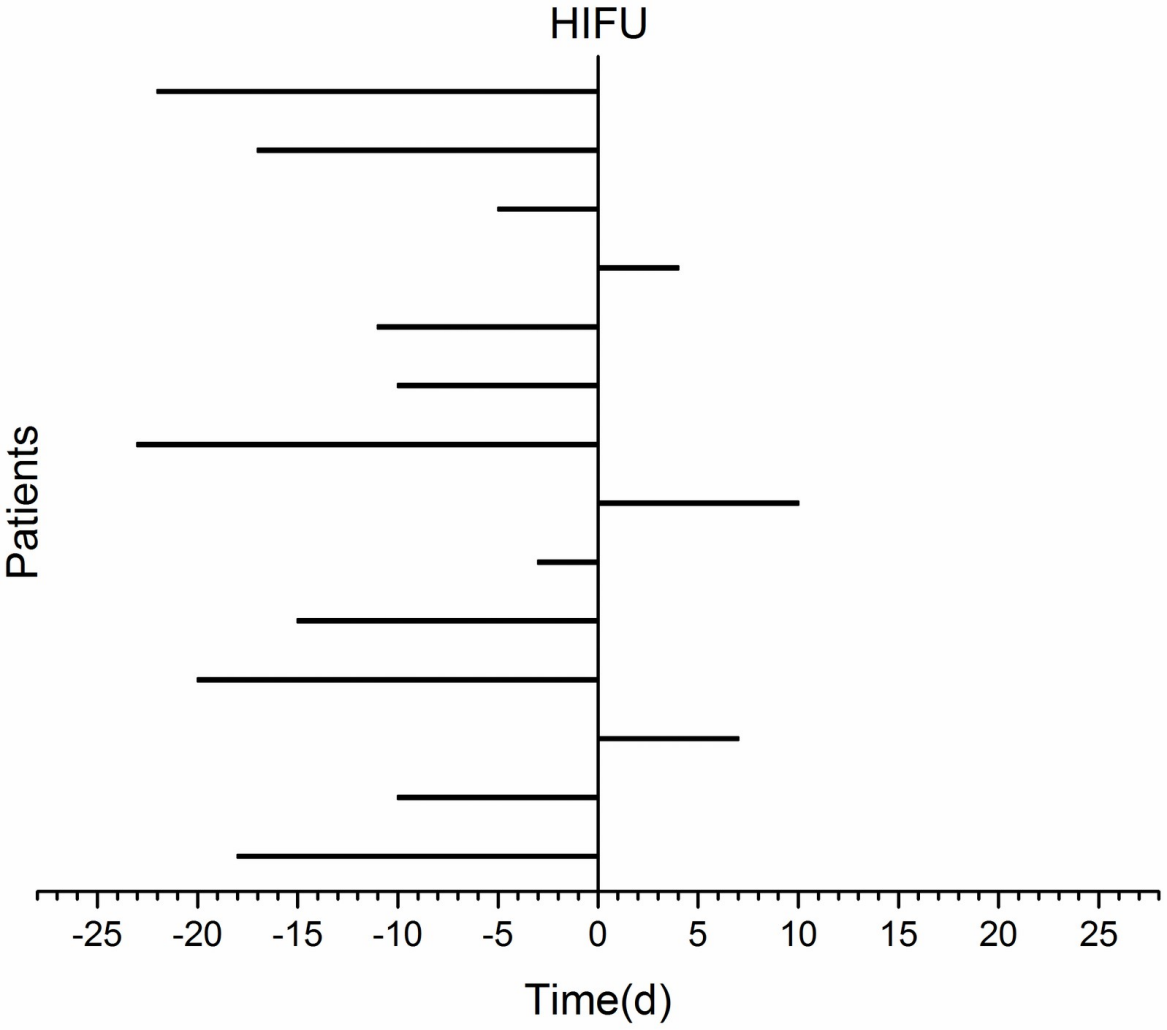
Specific time of immunotherapy.

Fourteen patients were included in the study, five men and nine women, with a median age of 63 years (range: 35–84 years) (Fig 1). Median follow-up period was 9 months (range: 3–21). No patients were lost to follow-up throughout the trial period. Table 1 described the clinical characteristics of the study population. Immunotherapy was performed within 1 month before and after HIFU ablation. All patients chose combination immunotherapy, and the immunotherapies selected for this trial are all anti-PD-1 agents manufactured in China. Most of the immunotherapy was prior to HIFU ablation, and only 3 patients added immunotherapy after HIFU ablation. Sintilimab lnjection was chosen in 6 cases, Toripalimab lnjection in 2 cases, Camrelizumab for lnjection in 2 cases, and Tislelizumab lnjection in 4 cases. Fig 2 illustrates the specific time of immunotherapy.

**Table 1:** Patients’ characteristics and baseline demographics.

Characteristics and demographics	No. (%)
**Patient demographics**	
Total No.	14
Age	
Median age(year)	63 (35–84)
>63	7 (50.0)
≤58	7 (50.0)
Sex	
Male	5 (35.7)
Female	9 (64.3)
**ECOG Score**	
0–1 point	8 (57.1)
2 points	6 (42.9)
**Clinical characteristics**	
Histologic diagnosis, No.	14
Lung cancer	3 (21.4)
Esophageal cancer	2 (14.3)
Hepatocellular carcinoma	2 (14.3)
Cholangiocarcinoma	2 (14.3)
Cervical cancer	1 (7.1)
Pancreatic cancer	1 (7.1)
Nasopharyngeal carcinoma	1 (7.1)
Gallbladder cancer	1 (7.1)
Sigmoid colon cancer	1 (7.1)
**No. of treated lesions**	
1	10 (71.4)
2	3 (21.4)
3	1 (7.1)

Table 2 demonstrates the supplementary clinical features of patients with LM. The mean body mass index was 23 (range: 15–28). The mean lesion volume was 179.9 cm3, with a maximum volume of 733.1 cm3. The clinical symptoms at baseline included abdominal pain in 7 patients (50.0%), abdominal distention in 3 patients (21.4%), and asymptomatic in 4 patients (28.6%). With reference to pre-HIFU and post-HIFU QOL quality of life (QOL) scores, there was no reduction in patients’ quality of life after HIFU. A total of seven patients were still alive after 12 months of follow-up. (Table 2).

**Table 2:** Supplementary baseline for clinical characteristics of patients with liver metastases.

Case	Age	BMI	Primary tumor	histologic subtypes	Tumor volume (cm^3^)	Symptoms before HIFU	Pre-HIFU QOL score	Post-HIFU QOL scores	Status
1	35	15	Cervical cancer	Adenosquamous carcinoma	393.3	Abdominal distention	70	70	Deceased
2	55	27.6	Lung cancer	Poorly differentiated adenocarcinoma	26.3	Asymptomatic	90	90	Surviving
3	46	25.1	Pancreatic cancer	Poorly differentiated adenocarcinoma	44.5	Abdominal pain	90	90	Deceased
4	74	21.5	Esophageal cancer	Poorly differentiated squamous cell carcinoma	58.5	Asymptomatic	80	90	Deceased
5	54	20.4	Nasopharyngeal carcinoma	Moderately-poorly differentiated squamous cell carcinoma	117.7	Abdominal pain	90	90	Deceased
6	58	20.4	Cholangiocarcinoma	Poorly differentiated adenocarcinoma	136.4	Abdominal pain	90	90	Deceased
7	37	24.2	Gallbladder cancer	Poorly differentiated adenocarcinoma	733.1	Abdominal pain	80	80	Deceased
8	73	22.5	Esophageal cancer	Moderately differentiated squamous cell carcinoma	4.5	Abdominal pain	80	80	Surviving
9	75	27.6	Lung cancer	Moderately-poorly differentiated squamous cell carcinoma	294.9	Abdominal pain	80	80	Surviving
10	56	24.3	Cholangiocarcinoma	Poorly differentiated adenocarcinoma	12.2	Abdominal pain	90	90	Surviving
11	69	23.0	Lung cancer	Moderately differentiated squamous cell carcinoma	16.2	Asymptomatic	90	90	Surviving
12	68	22.8	Sigmoid colon cancer	Moderately differentiated adenocarcinoma	259.0	Abdominal distention	90	90	Deceased
13	69	24.0	Hepatocellular carcinoma	Hepatocellular	57.0	Asymptomatic	90	90	Surviving
14	84	22.9	Hepatocellular carcinoma	Hepatocellular	364.8	Abdominal distention	80	90	Surviving

Among the 14 patients, there were a total of 19 distinct treatment sites: 10 patients received treatment at 1, 3 patients received treatment at 2, and 1 patient received treatment at 3 anatomical sites. All metastases are within the range of lesions treatable with HIFU ablation and have safe acoustic access. Treatment begins at the lowermost part of the target lesion, with 1 cm as the treatment unit. During the procedure, the patient’s vital signs and changes in the ultrasound image of the lesion are closely observed and the power and direction are adjusted in time. All patients chose to receive combined immunotherapy (the type of anti-PD1 was not defined).

### 3.2. HIFU treatment workflow feasibility

**Fig 3:**
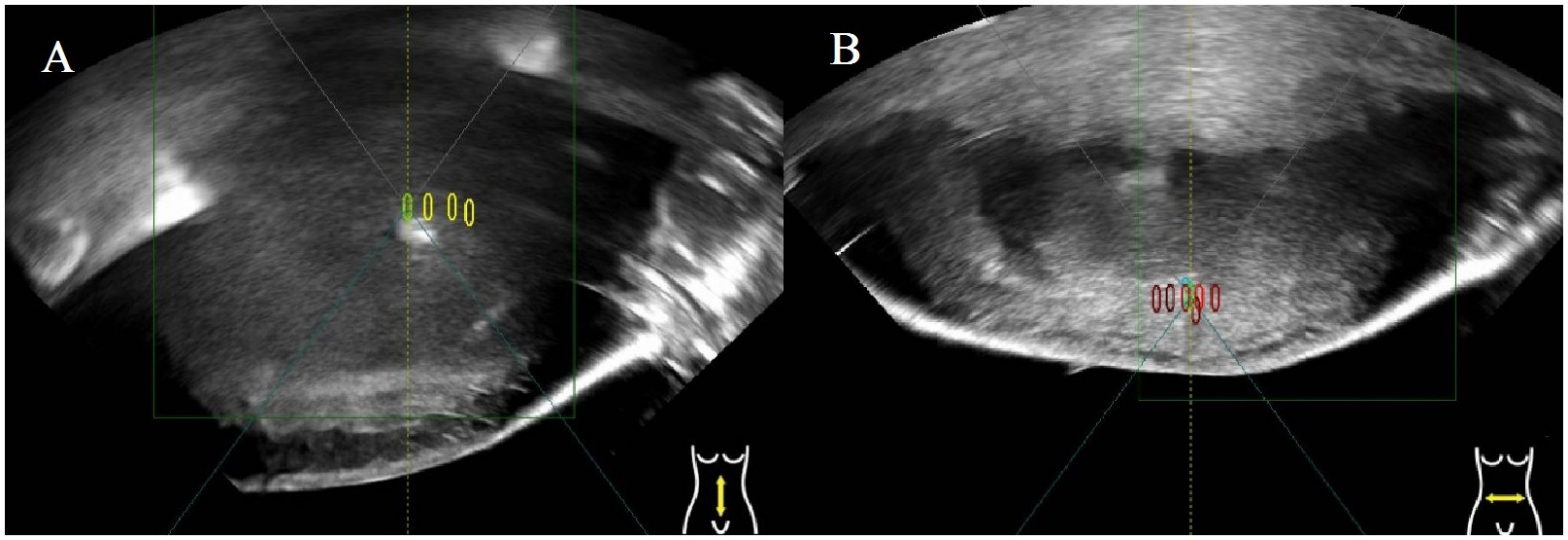
The real-time ultrasound image obtained from two patients with liver metastases. (A) shows the overall grayscale change: the gradient grayscale increases; (B) shows the Massive grayscale change: the area around the focal point quickly and noticeably grays and brightens grayscale We categorized grayscale changes into two types: overall grayscale changes and massive grayscale changes [1]. Overall grayscale change refers to a gradual increase in grayscale around the focal point, while massive grayscale change indicates an immediate and significant diffuse increase in grayscale in the focal area. However, there is no standard for grayscale changes in HIFU treatment for malignant tumors. Clinically, grayscale changes for malignant tumors are typically assessed based on the standard of grayscale changes in HIFU treatment for uterine fibroids.

Subjects complete at least two cycles for HIFU ablation combined with immunotherapy, and gray changes grayscale gray were observed in the target lesions after HIFU ablation (Fig 3). The workflow for HIFU ablation of liver metastases were shown in Table 3. HIFU, as a non-invasive treatment method, uses the thermal, cavitation and mechanical effects of ultrasound to achieve coagulative necrosis of tumours. After HIFU and also at the time of the first efficacy assessment(after 2 cycles of immunotherapy), CT examination showed that non-perfused areas were observed in all the 19 treated lesions. And the average NPV ratio was 77% (range: 58–88%), which can be assessed as a relatively satisfactory ablation. There were no equipment-related problems or delays in treatment with the HIFU treatment.

**Table 3:** Workflow for HIFU treatment of liver metastases.

Case	No. of treated lesions	Average power (w)	Treatment Time (min)	Sonication time (s)	Treatment time intensity (s/h)	Therapeutic Dose (J)	NPV ration	Gray scale change
1	1	400	240	2999	749.8	1199600	88	Massive grayscale changes
2	1	300	90	421	280.7	12000	64	Overall grayscale change
3	2	380	160	1199	449.6	467800	72	Massive grayscale changes
4	3	350	100	740	444	283800	58	Overall grayscale change
5	1	350	90	606	404	240000	76	Overall grayscale change
6	1	398	65	600	553.9	238800	86	Overall grayscale change
7	2	363	250	1986	476.6	726350	68	Massive grayscale changes
8	1	250	120	781	390.5	195250	80	Massive grayscale changes
9	1	400	290	2605	538.9	1042000	88	Overall grayscale change
10	1	399	150	1017	406.8	405300	80	Overall grayscale change
11	1	363	160	1157	433.9	420050	74	Overall grayscale change
12	2	390	195	3600	1107.7	727100	76	Massive grayscale changes
13	1	343	285	1680	353.7	890950	80	Overall grayscale change
14	1	344	210	3000	857.1	571950	86	Massive grayscale changes

### 3.3 Adverse events

A total of 21 adverse events in 14 patients were considered "probably" or "definitely related" to HIFU combined with immunotherapy. Most AEs (11 out of 21) were related to pain in the treatment area, with three of these reported as Grade 2 pain (Table 4), with no significant AEs identified. Other recorded AEs consisted of 4 cases of fatigue (28.6%), 3 cases showed rash (21.4%), and 1 case showed long-term skin discoloration (Grade 1). Most AEs were found to be reversible. There were an absence of treatment-related deaths or treatment-related grade 4 adverse reactions in this study.

**Table 4:** Adverse events (definitely, possibly, or probably) associated with HIFU treatment and immunotherapy (n = 14).

Adverse events	Patient, No. (%)
**Acute events (≤3 mo post-treatment)**	
Skin burn	none (0)
Surrounding structure injury	none (0)
Intestinal injury	none (0)
Pain in treatment area	
grade 1	8 (57.1)
grade 2	3 (21.4)
grade 3	none (0)
grade 4	none (0)
Nerve injury	none (0)
Fatigue (grade 1)	4 (28.6)
Myocarditis	none (0)
Rash (grade 1)	3 (21.4)
Pneumonitis	none (0)
**Long term events (>3 mo post-treatment)**	
Skin discoloration (grade 1)	1 (7.1)
Endocrine dysfunctions	none (0)
Liver dysfunction/failure (clinical)	none (0)

As shown in Table 5, HIFU relieved the patients’ pain, with five of the seven patients with abdominal pain receiving pain relief after HIFU treatment, although one of them had increased pain (case 10). Pain scores increased slightly between 3 and 12 months after HIFU. Two patients did not complain of pain through follow-up period.

**Table 5:** Recording of pain scores.

Case	Before	2-month	4-month	6-month	8-month	10-month	12-month
1	0	0	3	Deceased			
2	0	0	0	0	0	0	0
3	2	1	Deceased				
4	0	0	0	0	Deceased		
5	2	2	1	1	0	1	Deceased
6	4	1	1	1	3	Deceased	
7	4	1	2	3	Deceased		
8	3	1	1	1	0	0	0
9	2	1	1	0	0	0	0
10	4	4	5	5	5	5	4
11	0	0	1	0	0	0	0
12	0	0	1	Deceased	0	0	
13	0	0	1	1	1	3	3
14	0	0	0	2	2	4	3

### 3.4. Efficacy

**Fig 4:**
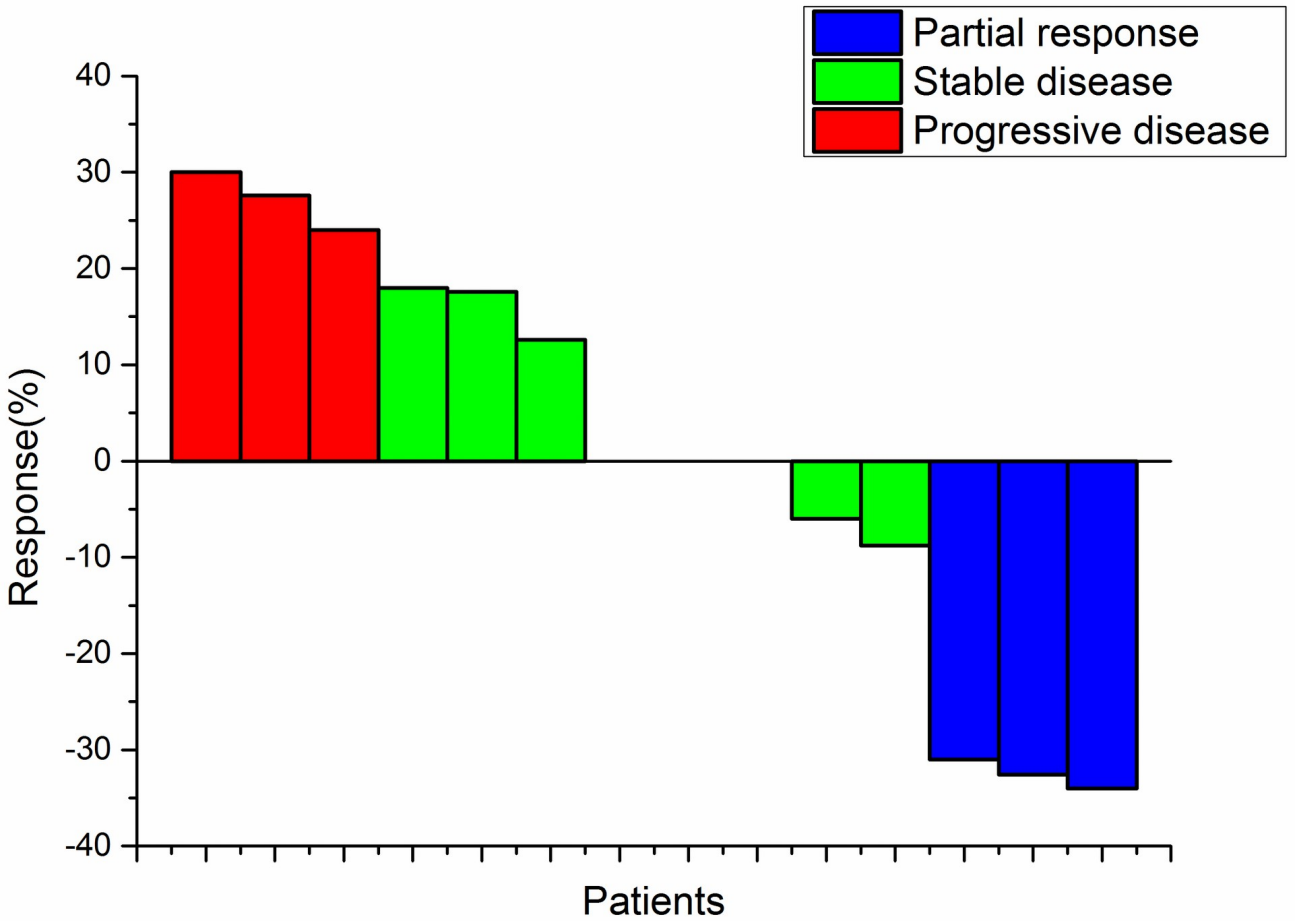
Waterfall plot for the optimal percentage change in all target lesions.

Before the cutoff date, no patients in the trial had achieved complete response (CR) and three patients (21.4%) showed disease progression after 2 cycles of combined therapy. However, eight patients (57.2%) achieved stable disease (SD) and three patients (21.4%) achieved partial response (PR). The objective response rate(ORR) and disease control rate(DCR) were 21.4% and 78.6%, respectively (Table 6). For these 14 patients who had at least one post-baseline efficacy assessment, a waterfall plot of the optimal percentage change in all target lesions was plotted (Fig 4).

**Table 6:** Assessment of best overall response following HIFU ablation combined with immunotherapy according to RESIST 1.1.

Tumor Response	Patient, No. (%)
Complete Response	0
Partial Response	3(21.4%)
Stable Disease	8(57.2%)
Disease Progression	3(21.4%)
Objective Response	3(21.4%)
Disease Control	11(78.6%)

## 4. Discussion

In this study, we investigated a novel combination therapy, systemic ICI-based immunotherapy, combined with local HIFU ablation in 14 patients with advanced LM. Notably, five patients experienced post-intervention pain relief, indicating the safety and clinical feasibility of combining the two treatments for LM.

Given the unique anatomy of the liver, which contributes to the expansion of local metastases and poor effectiveness to immunotherapy in LM patients, the combination of HIFU treatment and immunotherapy may achieve complementary results [51]. Moreover, HIFU ablation has shown to have immunomodulatory effects and can produce distinctive tumour fragments, inducing local inflammation with significant dendritic cell infiltration and enhancing dendritic cell-induced T-cell activation [42, 52]. Previous studies have shown that HIFU ablation systematically affects the secretion of immune anti-tumour factors such as IL-12 and IFN, and increases the number of mature dendritic cells through tumor lysates caused by HIFU ablation, which induces tumour cells apoptosis and intra-tumoural macrophages and lymphocytes infiltration [52, 53]. Joiner et al. indicated that focused ultrasound treatment caused direct tumour damage and altered macrophages and T cells in the tumour microenvironment 2 days after treatment; however, most of these effects will fade after day 15 of focused ultrasound treatment, illustrating the need for combination immunotherapy [54]. Various preclinical laboratory studies and clinical trials have shown that HIFU focused ultrasound enhanced an effective immune responses. The combination with immunotherapy should be more complementary to enhance the effectiveness of anti-tumour therapy [55–59].

This is the first prospective study of HIFU ablation combined with immunotherapy for liver metastases, as far as we know. Throughout the trial, each patient was effectively followed up, with no lost cases and median follow-up time of 9 (range 3–21) months. All metastases have a safe ultrasound pathway and predefined treatment field size and are within the range of lesions treatable with the HIFU technology. The average NPV(Non-Perfusion Volume) ratio was 77% (range: 58–88%), which can be assessed as a relatively satisfactory ablation. With reference to pre-HIFU and post-HIFU QOL scores, there was no reduction in patients’ quality of life after HIFU. Immunotherapy within 1 month before and after HIFU ablation was considered a combination of both treatments. Given the absence of literature comparing the efficacy and side effects of different immunological drugs in patients with liver metastases, we refrained from selecting a specific immune checkpoint inhibitor. Combination immunotherapy (anti-PD-1 agents manufactured in China) was chosen for all patients, and most immunotherapy was administered pre-HIFU ablation, with only three patients receiving additional immunotherapy post-HIFU ablation. While there are clinical trials investigating the use of HIFU for immunomodulation of malignant tumors, none have specifically examined the combination of HIFU with immunotherapy [50]. Based on relevant preclinical research, the timing of immunotherapy and HIFU treatment is not rigidly defined [52–54]. The researchers did not find any patients with skin burns during the trial. No major short-or long-term complications occurred. Most AEs (11 of 21) were related to pain in the treatment area, with 3 consisting of pain Grade 2. Other common AEs were fatigue (4 [28.6%]), rash (3 [21.4%]), and 1 case of long-term skin discoloration (Grade 1). Most AEs were found to be reversible. There were an absence of treatment-related deaths and treatment-related grade 4 adverse reactions in this study. Toxicity was manageable. Throughout the trial, patients were expected to survive for more than 3 months (the earliest deaths in Table 5 occurred at approximately 4 months). It is worth noting that the local safety and time to adverse events of HIFU is shorter compared to the systemic safety and time to adverse events of immunotherapy, which occurs approximately six months after immunotherapy [60–62]. Therefore, the number of deaths does not significantly impact the conclusions drawn from our study.

To better reflect the efficacy of HIFU ablation combined with immunotherapy, all target lesions (including measurable primary lesions, hepatic metastases, and remaining metastatic site lesions) were measured by computed tomography (CT) imaging and initial baseline conditions were recorded. For all 14 patients who had at least one post-baseline efficacy assessment, a waterfall plot of the optimal percentage change in all target lesions was plotted. Before the cutoff date, no patients in the trial had achieved complete response (CR) and three patients (21.4%) showed disease progression after 2 cycles of combined therapy. However, eight patients (57.2%) achieved stable disease (SD) and three patients (21.4%) achieved partial response (PR). The objective response rate(ORR) and disease control rate(DCR) were 21.4% and 78.6%, respectively (Table 6). A total of seven patients were still alive after 12 months of follow-up. As of data collected on 1 January 2023, one patient remained in a partial response (PR) state.

The strengths of this study are the ability to realise the needs of the clinicians and the patients, the unique and novel prospective design, the tracking of AE through follow-up, and the detailed workflow records and analysis. There were two limitations of the study. Firstly, patients’ long-term survival was not determined. Secondly, T cell infiltration at the HIFU-treated metastatic sites was not evaluated and changes in peripheral blood lymphocyte subsets were not regularly monitored. Therefore, the systemic immune cell changes, tumour microenvironment and long-term survival after HIFU combined with immunotherapy for LM need to be further investigated.

## 5. Conclusions

HIFU ablation combined with immunotherapy relieved tumor-related pain and prevented further local and systemic tumor growth to some extent. Based on these preliminary results, our prospective study confirms that HIFU combined with immunotherapy is clinically feasible and safe for LM patients. Future research should focus on addressing the limitations of the study, particularly by conducting longitudinal studies to assess the long-term survival outcomes of patients receiving combined HIFU and immunotherapy for LM. Additionally, investigating the immune response dynamics, tumor microenvironment changes, and identifying predictive biomarkers will contribute to optimizing treatment strategies and improving patient outcomes. Comparative studies against standard treatment modalities and optimization of treatment protocols are also essential for enhancing treatment efficacy and minimizing adverse effects in patients with LM.

## Supporting information

S1 Checklist TREND statement checklist. (PDF)

S1 File (DOC)

S2 File (DOCX)
