# Supplementary material for: High-intensity focused ultrasound ablation combined with immunotherapy for treating liver metastases: A prospective non-randomized trial
Source: PLoS One. 2024 Jul 5;19(7):e0306595. doi: 10.1371/journal.pone.0306595 (PMC11226133; doi:10.1371/journal.pone.0306595)
Supplement: S1 File — (DOC) [file pone.0306595.s002.doc]

内 部 资 料

注 意 保 密

**生物医学伦理研究方案**

**（干预性临床研究）**

**HIFU联合PD-1抑制剂治疗晚期恶性肿瘤I/II期临床研究方案**

研究单位：绵阳市中心医院

项目负责人：杜小波

承担科室：肿瘤科

联系电话：13550282229

组长单位：无

参加单位：无

研究年限：2020年10月－ 2023年02月

版本号：V1.0

版本日期：2020年09月24日

方 案 摘 要

| 研究设计  (可多选) | □ 病例对照研究 √ 队列研究 □ 横断面研究  □ 随机对照研究 □ 应用盲法 □ 其他： |
| --- | --- |
| 研究类型  （请根据项目类型勾选） | **（A类：高风险）**  □ 基因编辑研究  □ 细胞治疗研究  □ 植入性医疗器械研究（含3D打印）  □ Ⅲ类临床新技术（安全性、有效性确切，技术难度大、风险高）  □ 特殊人群研究（儿童、孕妇、智力低下者、精神障碍受试者等）  □ 超药物说明书研究（□超适应症 □超给药途径 □超剂量 □超年龄  □超禁忌症 □超人群 □其他，请说明： ）  □ 超器械说明书研究（□超适应症 □使用范围 □超禁忌症 □超人群  □其他，请说明： ）  □ 其他（研究者判定，请说明： ）  **（B类：中风险）**  □ 上市后生物制剂研究（预防用和治疗用）  □ 上市后治疗性疫苗研究  □ 上市后罕见病药物研究  √ Ⅱ类临床新技术（安全性、有效性确切，有一定技术难度，有一定医疗风险和伦理风险）  □ 其他（研究者判定，请说明： ）  **（C类：低风险）**  □ 已上市5年药物研究（包括化药、仿制药等）  □ 已上市器械研究（含AI，影像软件）  □ Ⅰ类临床新技术（安全性、有效性确切，技术难度低、几乎不存在伦理风险的医疗技术）  □ 其他（研究者判定，请说明： ） |
| 病例总数 | 27 |
| 风险/受益分析 |  |
| 风险判断 | □不大于最小风险 √大于最小风险  最小风险：指试验中预期风险的可能性和程度不大于日常生活、或进行常规体格检查或心理测试的风险 |
| **研究期限** | 2020 年 10月 01 日 至 2023年 02月01日 |

一、研究背景

正常人体免疫系统能够检测出各种各样的病原体和肿瘤细胞，并将它们与健康的宿主细胞区分开来。适应性免疫反应的诱导始于抗原呈递细胞（antigen-presenting cells，APCs），通过抗原呈递、细胞迁移，以及一系列免疫信号激活，将外源抗原呈递给T淋巴细胞以供其T细胞受体识别，诱导效应和记忆性CD4+和CD8+T淋巴细胞的分化。然后，这些细胞以协调一致的方式执行其效应器功能，以消除病原体感染的细胞或肿瘤细胞[1]。然而，在癌症患者中，淋巴细胞介导的免疫未能阻止原发性肿瘤的发展。APCs对肿瘤细胞的识别能力差，肿瘤细胞对这些APCs缺乏适当的激活，阻碍了有效免疫效应细胞的产生。此外，免疫抑制性细胞因子和抑制性肿瘤相关细胞的存在是肿瘤阻止有效CD8+细胞毒性T淋巴细胞（cytotoxic T lymphocytes ，CTLs）、CD4+辅助T细胞的诱导和建立的常见机制[2]。

在过去的几年里，通过T细胞检查点阻断来增强免疫系统正在成为一种对癌症患者具有临床益处的有效治疗方式。免疫检查点抑制剂如程序性死亡-1（Programmed death receptor-1，PD-1）和程序性死亡配体1（Programmed cell death 1 ligand 1，PD-L1）阻断抗体在临床试验中的成功极大地影响了几种恶性肿瘤的治疗策略[3-6]，免疫治疗方法也被认为是一种有前途的抗肿瘤策略。然而，免疫疗法的临床效果并不总是令人满意，既往临床研究报道的整体有效率仅为20%-30%[3-8]，它们的效果需要通过促进良好的免疫微环境来增强[9-10]。

高强度聚焦超声（high intensity focused ultrasound，HIFU）是我国自主研发的热消融技术，其原理是将超声波进行聚焦，利用声能在焦点处将温度提高到56℃到100℃之间，并引起肿瘤凝固性坏死[11]。除了热破坏外，HIFU还可利用（沸腾）组织碎裂术产生非热效应以破坏组织[12]。HIFU是迄今为止唯一一种完全无创的消融技术。与外科手术和其他消融技术相比，其优点是非侵入性，并且避免了因治疗操作导致的肿瘤转移，从而降低癌症患者的死亡率、复发率、住院时间、费用，并改善患者的生活质量。HIFU作为一种恶性肿瘤根治性和姑息性局部治疗手段，已越来越多地应用于临床治疗实体肿瘤，包括前列腺、肝脏、肾脏、乳腺、胰腺、骨骼和软组织[11]。更令人鼓舞的是，目前研究显示HIFU消融后残留在原位的肿瘤碎片可以作为免疫系统可用的肿瘤抗原 [13]。同时，肿瘤碎片可以用来制造一种原位肿瘤疫苗，这种疫苗能够刺激身体其他部位已经存在的（微）转移瘤的全身免疫反应 [14]。有研究显示，HIFU可以促进肿瘤内树突状细胞的浸润，并增强肿瘤细胞的抗原呈递过程[15]。Zhang[16]报道了HIFU消融后的肿瘤碎片能显著提高CTLs的数量和细胞毒性，并诱导未成熟DC活化。此外，在动物实验中显示，与未经治疗的对照组相比，热HIFU消融H22 HCC肿瘤导致CTLs的细胞毒性增加，同时IFN-γ和TNF-α分泌显著增加[17]。过继转移这些HIFU激活的CTLs在荷瘤小鼠的生存效益和肿瘤消退方面显示出强大的抗肿瘤免疫反应 [17]。 因此，HIFU用于增强免疫监测点抑制剂疗效被认为是一种可能。Silvestrini MT[18]等报道了HIFU联合PD-1抗体治疗恶性肿瘤的的一项基础研究，与单纯免疫治疗相比，在免疫治疗后1周进行HIFU消融治疗可以提高抗肿瘤效果。

尽管针对PD-1/ PD-L1免疫监测点的抑制剂被认为是一种有效、安全的治疗，但是当与HIFU联合时，需要评估新的治疗模式的安全性、有效性，因此本课题拟进行一项前瞻性、单中心、I/II期临床实验，通过纳入晚期首次行免疫治疗的患者，通过PD-1抗体联合HIFU，评估治疗的安全性、有效性。

二、研究目的

1. 主要目的：评估PD-1抑制剂联合HIFU手术治疗恶性肿瘤的安全性

2. 次要目的：评估PD-1抑制剂联合HIFU手术治疗恶性肿瘤的有效性

三、研究设计、方法与研究步骤

1. 研究设计

本研究为I/II期临床实验，主要评估HIFU联合PD-1抑制剂的安全性和有效性。主要纳入晚期拟行PD-1抑制剂治疗的晚期恶性肿瘤患者，同时患者体内有可供HIFU消融治疗的病灶。I期实验目标样本量为10名患者。主要终点是治疗毒性，次要终点是疗效。如果I期研究毒副反应可控，则继续入组至II期临床实验预期患者数量（再纳入17例，满足总共所需的27例患者），评估客观反应率（ORR），临床获益(定义为至少持续6个月的非消融病灶irCR/irPR/irSD，按irRECIST标准)、肿瘤无进展时间PFS(定义为从开始治疗到肿瘤进展或任意原因导致的死亡时间)、外周血淋巴细胞计数和免疫标记物变化、毒性反应。

试验的把握度为80%，α=0.05。据既往文献报道，单纯PD-1抑制剂治疗晚期恶性肿瘤的客观有效率为30%。预计在免疫治疗基础上联合HIFU治疗后，ORR可升至50%，经查表获得II期实验最佳样本量为27例（β=0.20， α=0.05）。报告毒性反应的发生率，计算并报告有临床反应的患者比例和95%置信区间。Kaplan-Meier方法分析疾病进展的速率和严重程度,方差分析用于比较治疗前、治疗中、治疗后的血液免疫指标变化。使用Fisher精确概率检验分析临床结果与血液指标间的相关性。

1. 研究方法

本研究采用I/II期单中心队列研究。I期实验目标样本量为10名患者。主要终点是治疗毒性，次要终点是疗效。如果I期研究毒副反应可控，则继续入组至II期临床实验预期患者数量（再纳入17例，满足总共所需的27例患者）。

3. 研究步骤


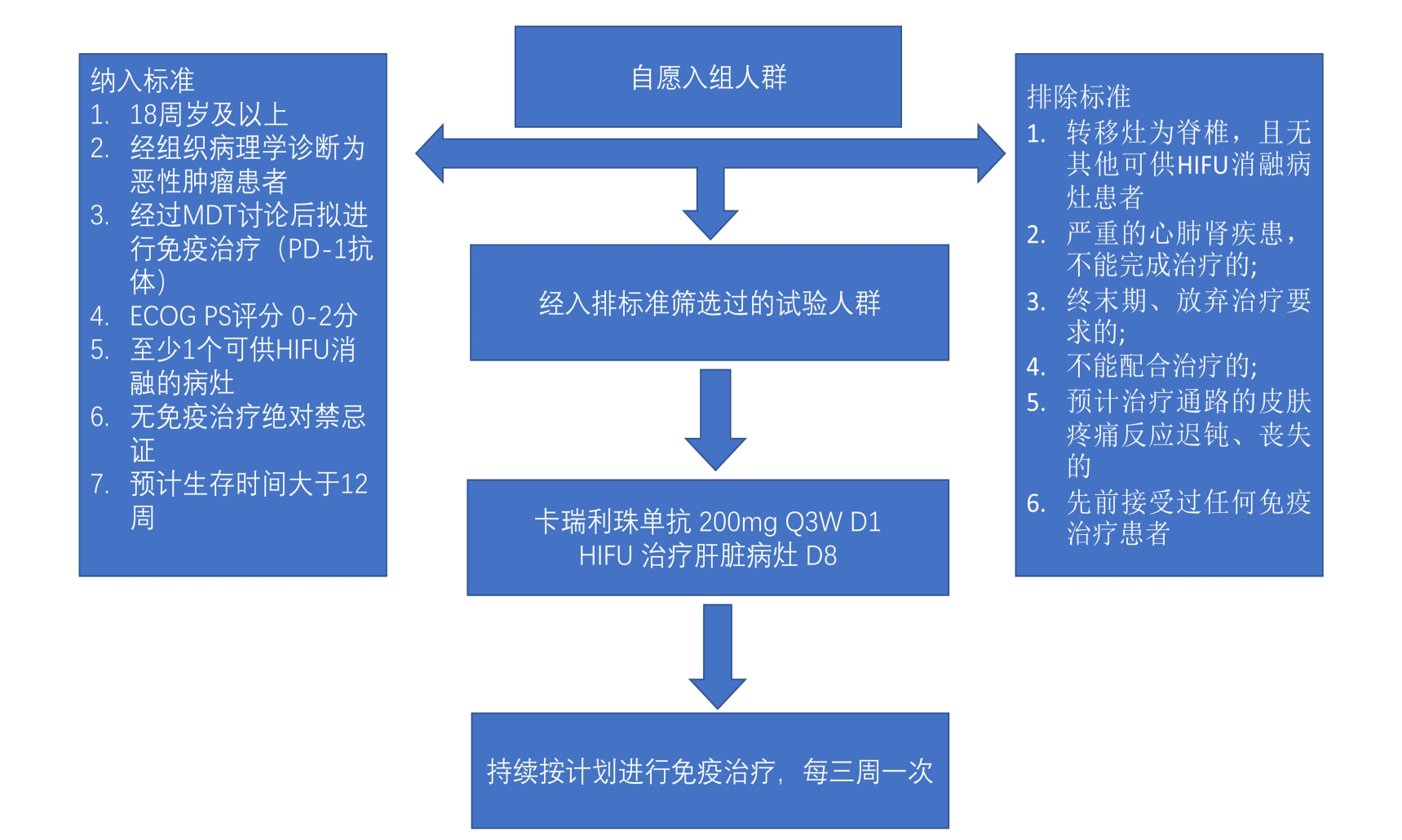


四、病例选择

1. 入选标准

1. 18周岁及以上
2. 经组织病理学诊断为恶性肿瘤患者
3. 经过MDT讨论后拟进行免疫治疗（PD-1抗体）
4. ECOG PS评分 0-2分
5. 至少1个可供HIFU消融的病灶，同时有≥1个额外的非连续性病变进行监测。
6. 无免疫治疗绝对禁忌证

2. 排除标准

1. 转移灶为脊椎或颅内，且无其他可供HIFU消融病灶患者
2. 严重的心肺肾疾患，不能完成治疗的;
3. 终末期、放弃治疗要求的;
4. 不能配合治疗的;
5. 预计治疗通路的皮肤疼痛反应迟钝、丧失的

先前接受过任何免疫治疗患者

3. 终止研究标准

如果超过2名患者出现不可控的治疗相关III-IV级毒性，本研究将提前结束。

1. 可供选择的其他诊疗方法

全身化疗，但疗效劣与本研究方案，且不良反应的发生率更高。

六、检测项目与检测时点

外周血淋巴细胞计数和免疫标记物变化：第一次给药前、HIFU治疗后、下一次给药前和最后一次给药后约3周取血样进行相关分析，淋巴细胞计数包括：总CD8+（CD3+CD8+）、CD4+T效应细胞（Teffs）（CD3+CD4+FOXP3-），CD4+T调节细胞（Treg）（CD3+CD4+FOXP3+CD127）；免疫标记物包括：4-1BB、OX40、LAG3、ICOS、GITR、CTLA4、TIM-3和PD1，免疫标记物表达以每个细胞群中表达细胞的百分比表示。

七、疗效评定标准

全身疗效评价时间为每两月进行一次影像学检查，采用irRECIST 标准评价评估疗效。

八、不良事件的观察、记录和处置

毒性反应评价标准为CTCAE v5.0标准，从治疗开始及进行连续性评估，至少评估至末次治疗后3月，出现不良反应应记录在CRF表中，并按照GCP不良事件与严重不良事件处理方案处理。

九、研究的质量控制与质量保证

本试验主要由绵阳市中心医院肿瘤科团队实施。肿瘤中心团队具备GCP（药物临床试验）资质和超声聚焦刀治疗恶性肿瘤经验。该课题负责人具备肿瘤学博士学位且是GCP的项目负责人，团队其他成员均具备GCP证书，能够保质保量完成临床试验。本项目具有重大意义，得到绵阳市医学会及绵阳市中心医院大力支持，副院长杜小波牵头，肿瘤科主导介入中心团队协助完成。

十、数据安全监查

临床研究将根据风险大小制定相应的数据安全监察计划。所有不良事件均详细记录，恰当处理并追踪直到妥善解决或病情稳定，按照规定及时向伦理委员会、主管部门、申办者和药品监督管理部门报告严重不良事件与非预期事件等；主要研究者定期对所有不良事件进行累积性回顾，必要时召开研究者会议评估研究的风险与受益；双盲试验必要时可以进行紧急揭盲，以确保受试者安全与权益。

十一、统计学处理

统计软件采用SPSS 22.0版本，试验的把握度为80%，α=0.05。据既往文献报道，单纯PD-1抑制剂治疗晚期恶性肿瘤的客观有效率为20%。预计在免疫治疗基础上联合HIFU治疗后，ORR可升至40%，经查表获得II期实验最佳样本量为27例（β=0.20， α=0.05）。报告毒性反应的发生率，计算并报告有临床反应的患者比例和95%置信区间。Kaplan-Meier方法分析疾病进展的速率和严重程度,方差分析用于比较治疗前、治疗中、治疗后的血液免疫指标变化。使用Fisher精确概率检验分析临床结果与血液指标间的相关性。

十二、临床研究伦理原则与要求

临床研究将遵循世界医学大会《赫尔辛基宣言》和中华人民共和国国家卫生和计划生育委员会《涉及人的生物医学研究伦理审查办法》等相关规定，具体落实知情同意，保护隐私，研究免费与补偿，控制风险，特殊受试者保护和研究相关损害的赔偿原则与要求。在研究开始之前，由伦理委员会批准该试验方案后才实施临床研究。每一位受试者入选本研究前，研究者有责任向受试者或/和其法定代理人完整、全面地介绍本研究的目的、程序和可能的风险，并签署书面知情同意书，应让受试者知道他们参加临床研究完全是自愿的，他们可以拒绝参加或在试验的任何阶段随时退出本研究而不会受到歧视和报复，其医疗待遇与权益不受影响。知情同意书应作为临床研究文件保留备查，切实保护受试者的个人隐私与数据机密性。

十三、研究进度

2020年12月-2021年10月：

完成I期临床实验所要求的10例晚期恶性肿瘤病人入组。

2021年11月-2022年2月：

完成I期临床实验所有入组病人随访，并完成HIFU联合PD-1抑制剂安全性评估。并开始撰写“HIFU联合PD-1抑制剂治疗晚期恶性肿瘤I期临床实验”SCI论文，开始投稿。

2022年03月-2022年10月：

如果I期实验证实HIFU联合PD-1抑制剂毒性反应可以耐受，则继续入组病人，并达到II临床实验既定的入组病例数量。

2022年11月-2023年02月：

完成所有病人随访，撰写SCI文章，并投稿和在线发表。

十四、参加人员

| **姓名** | **职称/专业** | | **任务** | **GCP培训证书** |
| --- | --- | --- | --- | --- |
| 杜小波 | 主任医师 | 项目负责人，统筹课题实施 | | 是 |
| 林斌伟 | 医师 | 患者入组及数据分析 | | 是 |
| 高峰 | 副主任医师 | 患者入组及数据分析 | | 是 |
| 廖东彪 | 主任医师 | HIFU治疗质量控制 | | 是 |
| 张羽 | 副主任医师 | 课题实施及质量控制 | | 是 |
| 冯岗 | 主任医师 | 课题实施及质量控制 | | 是 |
| 戴堂知 | 高级工程师 | 数据分析及JC200治疗及参数质量控制 | | 否 |
| 杨曦月 | 在读硕士研究生 | 病人随访 | | 否 |
| 杜欢 | 在读硕士研究 | 临床数据收集及统计分析 | | 否 |
| 范靓嘉 | 在读硕士研究生 | 临床数据收集及统计分析 | | 否 |

1. 主要参考文献

[1] van den Bijgaart RJ, Eikelenboom DC, Hoogenboom M, Fütterer JJ, den Brok MH, Adema GJ. Thermal and mechanical high-intensity focused ultrasound: perspectives on tumor ablation, immune effects and combination strategies. Cancer Immunol Immunother. 2017;66(2):247-258.

[2] Sharma P, Allison JP. Immune checkpoint targeting in cancer therapy: toward combination strategies with curative potential. Cell. 2015;161(2):205-214.

[3] Rittmeyer A, Barlesi F, Waterkamp D, Park K, Ciardiello F, von Pawel J, OAK Study Group et al. Atezolizumab versus docetaxel in patients with previously treated non-small-cell lung cancer (OAK): a phase 3, open-label, multicentre randomised controlled trial. Lancet. 2017;389:255–265.

[4] Reck M, Rodríguez-Abreu D, Robinson AG, Hui R, Csőszi T, Fülöp A, KEYNOTE-024 Investigators et al. Pembrolizumab versus chemotherapy for PD-L1-positive non-small-cell lung cancer. N Engl J Med. 2016;375:1823–1833.

[5] Borghaei H, Paz-Ares L, Horn L, Spigel DR, Steins M, Ready NE, et al. Nivolumab versus docetaxel in advanced nonsquamous non-small-cell lung cancer. N Engl J Med.

[6] Weber JS, D’Angelo SP, Minor D, Hodi FS, Gutzmer R, Neyns B, et al. Nivolumab versus chemotherapy in patients with advanced melanoma who progressed after anti-CTLA-4 treatment (CheckMate 037): a randomised, controlled open-label, phase 3 trial. Lancet Oncol. 2015;16:375–384.

[7] Hamanishi J, Mandai M, Ikeda T, Minami M, Kawaguchi A, Murayama T, et al. Safety and antitumor activity of anti-PD-1 antibody, nivolumab, in patients with platinum-resistant ovarian cancer. J Clin Oncol. 2015;33:4015–4022.

[8] Noguchi M, Moriya F, Koga N, Matsueda S, Sasada T, Yamada A, et al. A randomized phase II clinical trial of personalized peptide vaccination with metronomic low-dose cyclophosphamide in patients with metastatic castration-resistant prostate cancer. Cancer Immunol Immunother. 2016;65:151–160.

[9] Bahig H, Aubin F, Stagg J, et al. Phase I/II trial of Durvalumab plus Tremelimumab and stereotactic body radiotherapy for metastatic head and neck carcinoma. BMC Cancer. 2019;19(1):68.

[10] FElbers JBW, Al-Mamgani A, Tesseslaar MET, et al. Immuno-radiotherapy with cetuximab and avelumab for advanced stage head and neck squamous cell carcinoma: Results from a phase-I trial. Radiother Oncol. 2020;142:79-84.

[11] Wu F. High intensity focused ultrasound ablation and antitumor immune response. J Acoust Soc Am. 2013;134(2):1695-1701.

[12] Khokhlova VA, Fowlkes JB, Roberts WW, Schade GR, Xu Z, Khokhlova TD, Hall TL, Maxwell AD, Wang YN, Cain CA. Histotripsy methods in mechanical disintegration of tissue: towards clinical applications. Int J Hyperth. 2015;31:145–162.

[13] den Brok MH, Sutmuller RP, van der Voort R, Bennink EJ, Figdor CG, Ruers TJ, Adema GJ. In situ tumor ablation creates an antigen source for the generation of antitumor immunity. Cancer Res. 2004;64:4024–4029.

[14] Prise KM, O’Sullivan JM. Radiation-induced bystander signalling in cancer therapy. Nat Rev Cancer. 2009;9:351–360.

[15] Hu Z, Yang XY, Liu Y, et al. Investigation of HIFU-induced anti-tumor immunity in a murine tumor model. J Transl Med. 2007;5:34.

[16] Zhang Y, Deng J, Feng J, Wu F. Enhancement of antitumor vaccine in ablated hepatocellular carcinoma by high-intensity focused ultrasound. World J Gastroenterol. 2010;16:3584–3591.

[17] Xia JZ, Xie FL, Ran LF, Xie XP, Fan YM, Wu F. High-intensity focused ultrasound tumor ablation activates autologous tumor-specific cytotoxic T lymphocytes. Ultrasound Med Biol. 2012;38:1363–1371.

[18] Silvestrini MT, Ingham ES, Mahakian LM, et al. Priming is key to effective incorporation of image-guided thermal ablation into immunotherapy protocols. JCI Insight. 2017;2(6):e90521.
