# Supplementary material for: High-intensity focused ultrasound ablation combined with immunotherapy for treating liver metastases: A prospective non-randomized trial
Source: PLoS One. 2024 Jul 5;19(7):e0306595. doi: 10.1371/journal.pone.0306595 (PMC11226133; doi:10.1371/journal.pone.0306595)
Supplement: S2 File — (DOCX) [file pone.0306595.s003.docx]

**Biomedical Ethics Research Programme**

**(Interventional clinical research)**

**Protocol for a phase I/II clinical study of HIFU in combination with a PD-1 inhibitor for advanced malignancies**

Research unit: Mianyang Central Hospital

Project leader: Du Xiaobo

Department: Department of Oncology

Contact Tel: 13550282229

Team Leader: None

Participating units: None

Research Period：October 2020 - February 2023

Version number: V1.0

Version date: 24 September 2020

**Summary of the programme**

| Research Design  (multiple choice) | □ Case-control studies √ Cohort studies □ Cross-sectional studies  □ Randomised controlled studies □ Applied blinding □ Other： |
| --- | --- |
| Type of research  (Please tick according to the type of project) | **（A: high risk)**  □ Gene editing research  □ Cell therapy research  □ Implantable medical device research (including 3D printing)  □ Class III new clinical technology (exact safety and efficacy, technical difficulty and high risk)  □Special population studies (children, pregnant women, mentally  retarded, mentally handicapped subjects, etc.)  □ Superindication study (□Superindication □ Super route of  administration □ Superdose □ Superage) □ ultra-contraindication  □ ultra-population □ other, please specify: )  □ Ultra device specification study (□ Ultra indication □ Scope of use □ Ultra contraindication □ Ultra population) □ Other, please specify: )  □ Other (as determined by the researcher, please specify: )  **（B: medium risk)**  □ Post-market biologics studies (prophylactic and therapeutic)  □ Post-market therapeutic vaccine research  □ Post-market rare disease drug research  √ Class II new clinical technologies (with definite safety and efficacy, certain technical difficulties, and certain medical and ethical risks)□ Other (as determined by the researcher, please specify: )  **（C类：低风险）**  □ Drug research (including chemical drugs, generic drugs, etc.) that has been on the market for 5 years  □ Research on marketed devices (including AI, imaging software)  □ Class I new clinical technology (medical technology with definite safety and effectiveness, low technical difficulty and almost no ethical risk)  □ Other (as determined by the researcher, please specify: ) |
| Total number of cases | 27 |
| Risk/benefit analysis |  |
| risk judgement | □ not greater than minimal risk √ greater than minimal risk  Minimal risk: the likelihood and magnitude of the expected risk in a trial is no greater than the risk of going about one's daily life, or of performing a routine physical examination or psychological test |
| **Duration of the study** | 01 October 2020 to 01 February 2023 |

1. Background to the study

The normal human immune system detects a wide variety of pathogens and tumour cells and distinguishes them from healthy host cells. The induction of an adaptive immune response begins with antigen-presenting cells (APCs), which, through antigen presentation, cell migration, and activation of a range of immune signals, present exogenous antigens to T-lymphocytes for recognition by their T-cell receptors, inducing the differentiation of effector and memory CD4+ and CD8+ T-lymphocytes. These cells then perform their effector functions in a coordinated manner to eliminate pathogen-infected cells or tumour cells [1]. However, in cancer patients, lymphocyte-mediated immunity fails to prevent the development of primary tumours.Poor recognition of tumour cells by APCs and lack of proper activation of these APCs by tumour cells prevent the generation of effective immune effector cells. In addition, the presence of immunosuppressive cytokines and suppressor tumour-associated cells is a common mechanism by which tumours prevent the induction and establishment of effective CD8+ cytotoxic T lymphocytes (CTLs , CTLs), CD4+ helper T cells [2].

Over the past few years, boosting the immune system through T-cell checkpoint blockade is emerging as an effective therapeutic modality with clinical benefits for cancer patients. The success of immune checkpoint inhibitors such as programmed death receptor-1 (PD-1) and programmed cell death 1 ligand 1 (PD-L1) blocking antibodies in clinical trials has greatly influenced the therapeutic strategies for several malignancies [3-6]. Immunotherapeutic approaches are also considered a promising anti-tumour strategy. However, the clinical outcomes of immunotherapies are not always satisfactory, with previous clinical studies reporting overall efficacy rates of only 20-30% [3-8], and their effects need to be enhanced by promoting a favourable immune microenvironment [9-10].

High intensity focused ultrasound (HIFU) is a thermal ablation technique developed independently in China, which is based on the principle of focusing ultrasound waves and using acoustic energy to raise the temperature at the focal point to between 56°C and 100°C and cause coagulative necrosis of the tumour [11]. In addition to thermal destruction, HIFU can also be used to produce a non-thermal effect using (boiling) tissue fragmentation to destroy tissue [12].HIFU is the only completely non-invasive ablation technique to date. Its advantages over surgical and other ablation techniques are that it is non-invasive and avoids tumour metastasis due to treatment manipulation, thus reducing mortality, recurrence, hospital stay, costs and improving the quality of life of cancer patients.HIFU has been increasingly used in the clinical treatment of solid tumours as a radical and palliative local treatment for malignant tumours, including prostate, liver , kidney, breast, pancreas, bone and soft tissue [11]. More encouragingly, current studies have shown that tumour fragments remaining in situ after HIFU ablation can serve as tumour antigens available to the immune system [13]. At the same time, tumour debris can be used to create an in situ tumour vaccine that stimulates systemic immune responses to (micro)metastases already present elsewhere in the body [14]. It has been shown that HIFU promotes the infiltration of dendritic cells within the tumour and enhances the antigen-presenting process of tumour cells [15].Zhang [16] reported that tumour debris ablated by HIFU significantly increased the number and cytotoxicity of CTLs and induced the activation of immature DCs. Furthermore, in animal experiments, it was shown that ablation of H22 HCC tumours by thermal HIFU resulted in increased cytotoxicity of CTLs along with a significant increase in the secretion of IFN-γ and TNF-α compared to untreated controls [17]. Overtransfer of these HIFU-activated CTLs showed potent anti-tumour immune responses in terms of survival benefit and tumour regression in hormonal mice [17]. Therefore, the use of HIFU to augment the efficacy of immune-monitoring site inhibitors has been suggested as a possibility.Silvestrini MT [18] et al. reported a basic study of HIFU combined with a PD-1 antibody for the treatment of malignancy, in which HIFU ablation treatment 1 week after immunotherapy improved antitumour efficacy compared with immunotherapy alone.

Although inhibitors targeting PD-1/PD-L1 immune monitoring sites are considered to be an effective and safe therapy, the safety and efficacy of the new treatment modality needs to be evaluated when combined with HIFU. Therefore, we propose to conduct a prospective, single-centre, phase I/II trial to evaluate the safety and efficacy of the treatment by enrolling advanced patients who are first-time immunotherapy patients, and then combining them with HIFU through PD-1 antibody. Evaluate the safety and efficacy of the treatment.

1. Purpose of the study

1. Primary objective: to assess the safety of PD-1 inhibitors combined with HIFU surgery in the treatment of malignant tumours

2. Secondary objective: to assess the efficacy of PD-1 inhibitors combined with HIFU surgery in the treatment of malignant tumours

1. Research design, methodology and research steps

1. Study design

This study is a phase I/II clinical trial to evaluate the safety and efficacy of HIFU in combination with PD-1 inhibitors. Patients with advanced malignant tumours proposed for PD-1 inhibitor treatment who also have lesions in their body that are amenable to ablative treatment with HIFU are primarily enrolled.The target sample size for the phase I trial is 10 patients. The primary endpoint is treatment toxicity and the secondary endpoint is efficacy. If the phase I study toxicity was manageable, enrolment was continued to the expected number of patients for the phase II clinical trial (17 more patients were enrolled to meet the total required 27 patients), assessing objective response rate (ORR), clinical benefit (defined as irCR/irPR/irSD of non-ablative lesions lasting at least 6 months, according to irRECIST criteria), tumour progression-free time PFS (defined as time from initiation of treatment to tumour progression or death from any cause), changes in peripheral blood lymphocyte counts and immune markers, and toxicity reactions.

The certainty of the trial was 80%, α=0.05. The objective effectiveness rate of PD-1 inhibitors alone in the treatment of advanced malignancies has been previously reported in the literature to be 30%. It was expected that the ORR could rise to 50% after combining HIFU therapy on top of immunotherapy, and the optimal sample size for the phase II trial was 27 cases (β=0.20, α=0.05), which was obtained by checking the table. The incidence of toxic reactions was reported, and the proportion of patients with a clinical response and 95% confidence intervals were calculated and reported.The Kaplan-Meier method was used to analyse the rate and severity of disease progression, and analysis of variance (ANOVA) was used to compare the changes in haematological immunological indices before, during and after treatment. Fisher's exact probability test was used to analyse the correlation between clinical outcomes and blood markers.

2.Research Methods

This study was a phase I/II single-centre cohort study.The target sample size for the phase I trial was 10 patients. The primary endpoint is treatment toxicity and the secondary endpoint is efficacy. If the Phase I study toxicity was manageable, enrolment was continued to the expected number of patients for the Phase II clinical trial (17 more were enrolled to fulfil the total required number of 27 patients).

3. Steps of the study


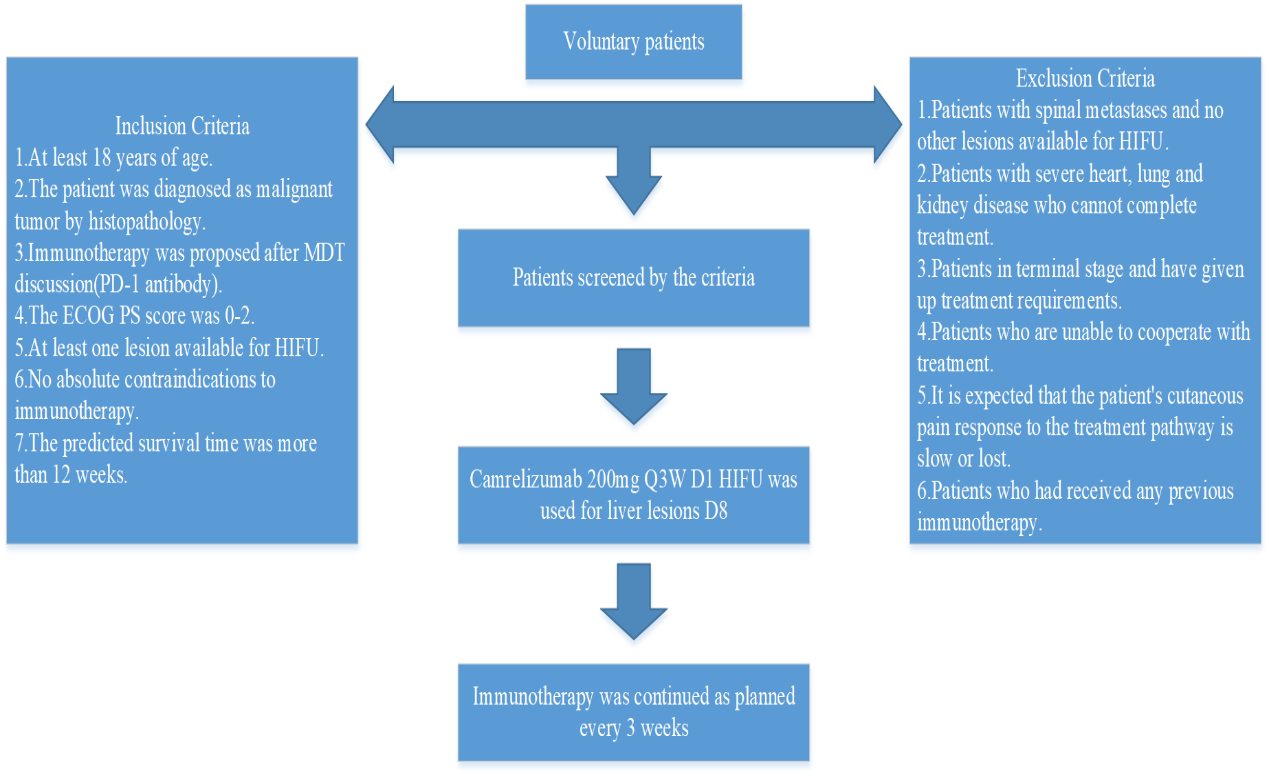


1. Case selection

1. Admission Criteria

A. 18 years old and above

B. Patients with malignant tumours diagnosed by histopathology

C. Proposed immunotherapy (PD-1 antibody) after MDT discussion

D. ECOG PS score of 0-2

E. at least 1 lesion available for HIFU ablation with ≥1 additional discontinuous lesion for surveillance

F. no absolute contraindications to immunotherapy

2. Exclusion criteria

A. patients with spinal or intracranial metastases and no other lesions available for HIFU ablation

B. Severe cardiopulmonary and renal disorders that prevent completion of treatment.

C. End-stage, abandonment of treatment requirements; D. Cannot cooperate with the treatment; and

D. Inability to co-operate with treatment; and

E. Blunted or loss of cutaneous pain response in the expected treatment pathway.

Patients who have received any prior immunotherapy

3. Criteria for study termination

This study will be terminated prematurely if more than 2 patients develop uncontrollable treatment-related Grade III-IV toxicity.

1. Alternative diagnostic and treatment options

systemic chemotherapy, but the efficacy was inferior to that of the present study protocol and the incidence of adverse effects was higher.

1. Test items and test points

Changes in peripheral blood lymphocyte counts and immune markers: blood samples were taken for correlation analyses before the first dose, after HIFU treatment, before the next dose, and approximately 3 weeks after the last dose; lymphocyte counts included: total CD8+ (CD3+CD8+), CD4+ T effector cells (Teffs) (CD3+CD4+FOXP3-), CD4+ T regulatory cells ( Treg) (CD3+CD4+FOXP3+CD127); immunomarkers included: 4-1BB, OX40, LAG3, ICOS, GITR, CTLA4, TIM-3, and PD1, and immunomarker expression was expressed as a percentage of expressed cells in each cell population.

1. Criteria for assessing efficacy

Systemic efficacy was evaluated at bimonthly imaging visits, and efficacy was assessed using irRECIST criteria evaluation.

1. Observation, recording and disposition of adverse events

Toxicity reactions were evaluated according to the CTCAE v5.0 criteria from the beginning of treatment and on a continuous basis, at least until 3 months after the last treatment, and the occurrence of an adverse reaction should be recorded in the CRF form and handled in accordance with the GCP Adverse Events and Serious Adverse Events protocol.

1. Quality control and quality assurance of research

This trial was mainly carried out by the oncology team of Mianyang Central Hospital. The oncology centre team has GCP (drug clinical trial) qualification and experience in ultrasound focused knife treatment of malignant tumours. The project leader has a PhD degree in oncology and is the project leader of GCP, and all other team members have GCP certificates and are capable of completing the clinical trial with quality and quantity. This project is of great significance and is strongly supported by Mianyang Medical Association and Mianyang Central Hospital. Vice President Du Xiaobo led the project, and the Oncology Department led the Interventional Centre team to complete the project.

1. Data security monitoring

A data safety monitoring programme will be developed for clinical studies according to the level of risk. All adverse events will be recorded in detail, handled appropriately and followed up until they are properly resolved or the condition is stabilised, and serious adverse events and unintended events will be reported to the Ethics Committee, the competent authority, the sponsor, and the drug regulatory authority in a timely manner in accordance with the regulations; the principal investigator will conduct a cumulative review of all adverse events on a regular basis, and convene a meeting of the investigators to assess the risks and benefits of the study when necessary; double-blind trials can be conducted when necessary. Emergency unblinding is carried out to ensure the safety and rights of the subjects.

1. statistical processing

The statistical software was SPSS version 22.0, and the certainty of the trial was 80%, α=0.05. According to previous literature, the objective effectiveness rate (ORR) for the treatment of advanced malignant tumours with PD-1 inhibitors alone was reported to be 20%. It was expected that the ORR could rise to 40% after combining HIFU treatment on top of immunotherapy, and the optimal sample size for the phase II trial was 27 cases (β=0.20, α=0.05) obtained by checking the table. The incidence of toxic reactions was reported, and the proportion of patients with a clinical response and 95% confidence intervals were calculated and reported.The Kaplan-Meier method was used to analyse the rate and severity of disease progression, and analysis of variance (ANOVA) was used to compare the changes in haematological immunological indices before, during and after treatment. Fisher's exact probability test was used to analyse the correlation between clinical outcomes and blood markers.

1. Ethical Principles and Requirements for Clinical Research

The clinical study will follow the relevant provisions of the Declaration of Helsinki of the World Medical Assembly and the Measures for Ethical Review of Biomedical Research Involving Human Beings of the National Health and Family Planning Commission of the People's Republic of China, which specify the implementation of the principles and requirements of informed consent, protection of privacy, research free of charge and compensation, control of risks, protection of special subjects, and compensation for research-related damages. Prior to the commencement of the study, the trial protocol was approved by the Ethics Committee before the clinical study was implemented. Before each subject is enrolled in the study, it is the responsibility of the investigator to provide the subject or/and his/her legal representative with a complete and comprehensive description of the purpose, procedures, and possible risks of the study, and to sign a written informed consent form, and to let subjects know that their participation in the clinical study is entirely voluntary, that they may refuse to participate or withdraw from the study at any time during any phase of the trial without discrimination or reprisal, and that their medical treatment and rights will not be affected. The informed consent form should be retained as a clinical research document for record-keeping purposes, and the privacy and confidentiality of the subjects' data should be effectively protected.

1. Research progress

December 2020-October 2021:

Completion of the required enrolment of 10 patients with advanced malignancies for the phase I clinical trial.

November 2021-February 2022:

Complete follow-up of all patients enrolled in the Phase I clinical trial and complete safety assessment of HIFU in combination with PD-1 inhibitors. Start to write the SCI paper "Phase I clinical trial of HIFU combined with PD-1 inhibitor in advanced malignant tumours" and start to submit the paper.

March 2022-October 2022: The study is expected to be completed by the end of this year:

If the Phase I trial confirms that the toxicity of HIFU in combination with PD-1 inhibitors is tolerable, patients will continue to be enrolled and the established number of cases enrolled in the Phase II trial will be reached.

Nov 2022 - Feb 2023:

Completion of all patient follow-ups and writing of SCI article for submission and online publication.

1. Participants

| **Name** | **Professional title/major** | | **Task** | **GCP training certificate** |
| --- | --- | --- | --- | --- |
| Xiaobo Du | chief physician | Project leader, co-ordinating the implementation of the project | | yes |
| Binwei Lin | resident physician | Patient enrolment and data analysis | | yes |
| Feng Gao | deputy chief physician | Patient enrolment and data analysis | | yes |
| Dongbiao Liao | chief physician | Quality control of HIFU treatment | | yes |
| Yu Zhang | deputy chief physician | Project implementation and quality control | | yes |
| Gang Feng | chief physician | Project implementation and quality control | | yes |
| Tangzhi Dai | senior engineer | Data analysis and quality control of JC200 treatments and parameters | | no |
| Xiyue Yang | resident | Patient follow-up and clinical data collection and statistical analysis | | yes |
| Huan Du | Master's studies in progress | clinical data collection and statistical analysis | | no |
| Jinjia Fan | Master's studies in progress | clinical data collection and statistical analysis | | no |

1. Main references

[1] van den Bijgaart RJ, Eikelenboom DC, Hoogenboom M, Fütterer JJ, den Brok MH, Adema GJ. Thermal and mechanical high-intensity focused ultrasound: perspectives on tumor ablation, immune effects and combination strategies. Cancer Immunol Immunother. 2017;66(2):247-258.

[2] Sharma P, Allison JP. Immune checkpoint targeting in cancer therapy: toward combination strategies with curative potential. Cell. 2015;161(2):205-214.

[3] Rittmeyer A, Barlesi F, Waterkamp D, Park K, Ciardiello F, von Pawel J, OAK Study Group et al. Atezolizumab versus docetaxel in patients with previously treated non-small-cell lung cancer (OAK): a phase 3, open-label, multicentre randomised controlled trial. Lancet. 2017;389:255–265.

[4] Reck M, Rodríguez-Abreu D, Robinson AG, Hui R, Csőszi T, Fülöp A, KEYNOTE-024 Investigators et al. Pembrolizumab versus chemotherapy for PD-L1-positive non-small-cell lung cancer. N Engl J Med. 2016;375:1823–1833.

[5] Borghaei H, Paz-Ares L, Horn L, Spigel DR, Steins M, Ready NE, et al. Nivolumab versus docetaxel in advanced nonsquamous non-small-cell lung cancer. N Engl J Med.

[6] Weber JS, D’Angelo SP, Minor D, Hodi FS, Gutzmer R, Neyns B, et al. Nivolumab versus chemotherapy in patients with advanced melanoma who progressed after anti-CTLA-4 treatment (CheckMate 037): a randomised, controlled open-label, phase 3 trial. Lancet Oncol. 2015;16:375–384.

[7] Hamanishi J, Mandai M, Ikeda T, Minami M, Kawaguchi A, Murayama T, et al. Safety and antitumor activity of anti-PD-1 antibody, nivolumab, in patients with platinum-resistant ovarian cancer. J Clin Oncol. 2015;33:4015–4022.

[8] Noguchi M, Moriya F, Koga N, Matsueda S, Sasada T, Yamada A, et al. A randomized phase II clinical trial of personalized peptide vaccination with metronomic low-dose cyclophosphamide in patients with metastatic castration-resistant prostate cancer. Cancer Immunol Immunother. 2016;65:151–160.

[9] Bahig H, Aubin F, Stagg J, et al. Phase I/II trial of Durvalumab plus Tremelimumab and stereotactic body radiotherapy for metastatic head and neck carcinoma. BMC Cancer. 2019;19(1):68.

[10] FElbers JBW, Al-Mamgani A, Tesseslaar MET, et al. Immuno-radiotherapy with cetuximab and avelumab for advanced stage head and neck squamous cell carcinoma: Results from a phase-I trial. Radiother Oncol. 2020;142:79-84.

[11] Wu F. High intensity focused ultrasound ablation and antitumor immune response. J Acoust Soc Am. 2013;134(2):1695-1701.

[12] Khokhlova VA, Fowlkes JB, Roberts WW, Schade GR, Xu Z, Khokhlova TD, Hall TL, Maxwell AD, Wang YN, Cain CA. Histotripsy methods in mechanical disintegration of tissue: towards clinical applications. Int J Hyperth. 2015;31:145–162.

[13] den Brok MH, Sutmuller RP, van der Voort R, Bennink EJ, Figdor CG, Ruers TJ, Adema GJ. In situ tumor ablation creates an antigen source for the generation of antitumor immunity. Cancer Res. 2004;64:4024–4029.

[14] Prise KM, O’Sullivan JM. Radiation-induced bystander signalling in cancer therapy. Nat Rev Cancer. 2009;9:351–360.

[15] Hu Z, Yang XY, Liu Y, et al. Investigation of HIFU-induced anti-tumor immunity in a murine tumor model. J Transl Med. 2007;5:34.

[16] Zhang Y, Deng J, Feng J, Wu F. Enhancement of antitumor vaccine in ablated hepatocellular carcinoma by high-intensity focused ultrasound. World J Gastroenterol. 2010;16:3584–3591.

[17] Xia JZ, Xie FL, Ran LF, Xie XP, Fan YM, Wu F. High-intensity focused ultrasound tumor ablation activates autologous tumor-specific cytotoxic T lymphocytes. Ultrasound Med Biol. 2012;38:1363–1371.

[18] Silvestrini MT, Ingham ES, Mahakian LM, et al. Priming is key to effective incorporation of image-guided thermal ablation into immunotherapy protocols. JCI Insight. 2017;2(6):e90521.

1. Deviations of the manuscript from this study protocol

(1) Because different primary malignancies are not always clinically appropriate for carelizumab and patients have the right to choose different immunological agents. Therefore, the initial designated immunological drug - carilizumab during the trial was changed to a clinically appropriate PD-1/PD-L1 inhibitor that was voluntarily selected by the patients. (2) The pre-trial design of this study was a phase I/II clinical trial to evaluate the safety and efficacy of HIFU in combination with a PD-1 inhibitor. However, the search of related literature during the trial found that there is no relevant study of HIFU combined with immunotherapy after the present, so the focus should not be on the validity of phase II, but more on the safety of phase I. The sample size of the trial should not follow Simon's phase II clinical trial design sample size of 27 cases. Instead, a prospective non-randomised phase I clinical trial should be designed.
